# Attuning Percutaneous Coronary Interventional Quality Metrics and Practice Modification∗

**DOI:** 10.1016/j.jacasi.2024.01.003

**Published:** 2024-02-20

**Authors:** Lloyd W. Klein

**Affiliations:** Cardiology Division, University of California, San Francisco, California, USA

**Keywords:** benchmarking, feedback, percutaneous coronary intervention, quality indicator

**Figure:**
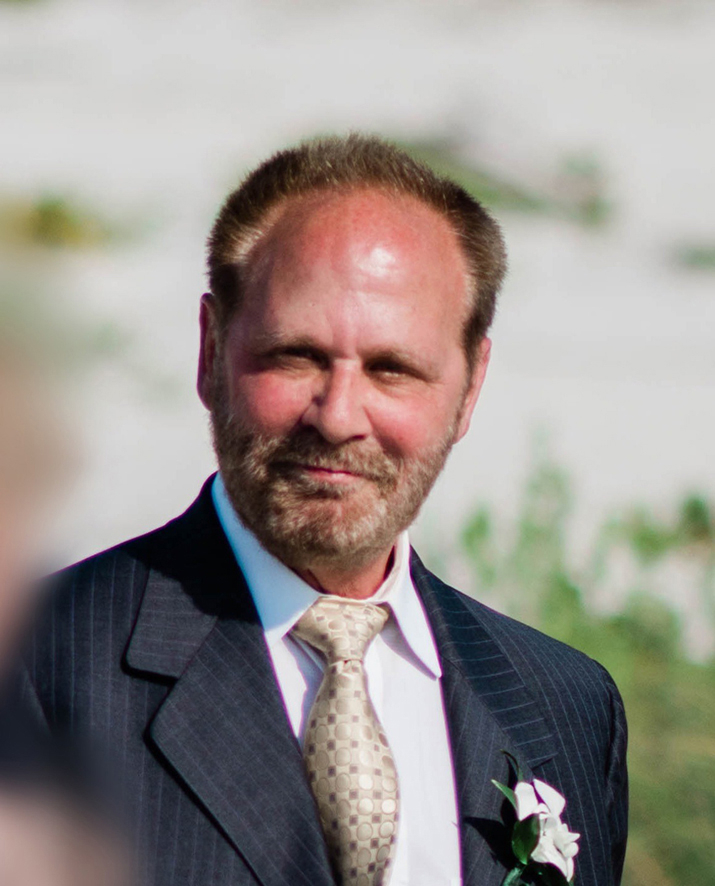

An accurate quality assessment process is essential to ensure superior performance of invasive cardiovascular procedures. Effective programs commit to pinpointing their flaws and refining implementation to achieve excellent outcomes, creating a culture of quality. The delivery of optimal clinical benefit requires an ongoing self-assessment structure comparing actual results to accepted benchmarks, with timely modification of practices when deficiencies are identified. The critical quality elements include adhering to evidence-driven case selection, ensuring proficient technical performance, and monitoring clinical outcomes.^1^, ^2^, ^3^

Benchmarking refers to comparing a center's process and performance metrics to similar programs. The purposes are to analyze one’s performance objectively and to better understand how to become more successful by detecting problem areas. The expectation is that such comparisons will ultimately positively influence programmatic outcomes by motivating efforts to improve the systemic and individual weaknesses identified.

In this issue of *JACC: Asia*, Saito et al^4^ present an analysis of the impact of benchmarking outcomes in the J-PCI (Japanese PCI) registry. This nationwide database collects data from 1,194 member institutions that perform >90% of the percutaneous coronary intervention (PCI) procedures in Japan. Data collection is incorporated into the National Clinical Data System, which is a nationwide internet-based registry endorsed by the Japanese Association of Cardiovascular Intervention and Therapeutics. Over 3 years, 734,264 consecutive PCI procedures with complete data were enrolled. Seven widely recognized quality indicators for each institution were benchmarked against other programs. The 62.1% of institutions that reviewed this data, a median of 4 times, were more likely to be academic and high-volume centers compared with those institutions that did not review the data. In the first year, those centers that reviewed these indicators had a modest improvement in some outcomes compared with nonreviewing institutions, but after the first year, there was no difference. Disappointingly, the only improvements were a slight increase in pre-PCI stress testing and a minor 2.3% increase in radial artery access in the first year only; in-hospital mortality was unchanged. Such trivial benefits might suggest at first glance that the entire concept of benchmarking is a colossal waste of resources. The main takeaway message is that informing participating institutions of opportunities for improvement counterintuitively fails to produce measurable quality enhancement.

This study raises a multitude of intriguing questions about the nature of quality improvement and exactly what operators and institutions hope to gain by participating in these registries.^5^ Most academic institutions seek to demonstrate their superiority, and many private hospitals want to show that their work is as good and maybe better. All are therefore unprepared when benchmarking shows that clinical outcomes overall tend toward the mean; that case selection is the primary determinant of procedural benefits, not operator competence; and that most everyone has some area that can be improved. These are unexpected messages that participants are not disposed to accept. The discouraging results of the Japanese Registry also signal a serious problem with the design and utilization of quality assurance programs. When prospectively tested, benchmarking did not motivate long-term adjustment; almost 40% of the programs were not even interested enough to look.

To better attune benchmarking to quality improvement requires a consideration of the principal reasons for this disengagement. Myriad obstacles to change are encountered in clinical practice, and reluctance to acknowledge limitations characteristically overcomes the aspiration to be the best. Interventional programs are under external pressure to achieve several vital but frequently conflicting objectives, including easier access to care, greater profitability, cost containment, and patient satisfaction, all to be accomplished with low complication rates despite treating increasingly higher-risk cases. These worthy goals are often inherently incompatible with candid self-assessment and improvement. Therein lies the conundrum: benchmarking processes paradoxically become viewed as an impediment to practice. Time and intellect are then diverted from improving systems to achieve better results. Instead, effort is expended on devising workarounds to circumvent the accurate collection of information that might show their performance in a bad light.^1^, ^2^, ^3^^,^^5^

Strict adherence to evidence-based guidelines tends to reduce the number of cases and scope of indications. There is also a synergistic systems component to this concern: in practice environments, where volume strongly determines hospital revenue as well as individual compensation, aggressive interventionists are highly valued. Adherence to guidelines is not a fashionable goal, nor is striving for outstanding results: maximizing case volume without falling below a threshold of adequacy has been the modern ambition of many institutions.

In some practice situations, any below-average quality indicator result becomes weaponized against the leaders of the program, rather than a tool for their members’ self-improvement. In such milieus, the economic and political incentives to hide rather than face reality become determinative: exactly the opposite of its original intention. Public reporting amplifies these fears, with the added burden that its “consumers” are not exclusively prospective patients, who have limited choices because of travel constraints and insurance coverage, but include hospital administrators and insurance plans looking to reduce expenditures.^6^

Measuring quality as in-hospital or 30-day mortality leads to exactly the wrong effect. The factor most powerfully associated with mortality is not operator competence but the indication for the procedure, particularly the acuteness of presentation.^7^ The widespread custom of defining this outcome as quality is fallacious and obsolete; the lack of change in mortality in Saito et al^4^ may be related to increasing case complexity. Furthermore, process indicators, as shown in the Japanese Registry, do not necessarily impact mortality when evaluated outside clinical trials. Instead of providing an incentive to choose the highest risk cases with the most to gain by successful revascularization, benchmarking paradoxically tends to discourage high-acuity cases. This unintended consequence further feeds the narrative that the quality assessment process is itself a hurdle to providing quality care.

Developing and implementing a successful internal quality process begins by setting high performance standards and then effectively communicating necessary modifications to improve outcomes. Uncooperative reactions to perceived misjudgments of physician worth and lack of confidence in the process are modifiable if there is sufficient support from both administration and physician leaders to create a safe space for authentic self-evaluation. Assuring physicians that their participation in constructive quality improvement is valued is a decisive factor in negotiating change.^1^ Unless these social apprehensions are addressed and incentives are aligned, a quality evaluation program is limited in what it may achieve.^6^

The optimal method to ensure best practices starts before the patient enters the catheterization laboratory; is evaluated prospectively, not postprocedure; and includes actionable endpoints. A portfolio of better indicators of quality has been proposed^2^ but has not been adopted because its establishment would require a serious commitment of effort and cost to update data collection mechanisms. Another hesitation has been that improving utilization and outcomes is desirable but not at the expense of increased cost and reduced revenue.

Asking the wrong question predictably leads to the wrong answer, and that is why the study of Saito et al^4^ needs to be taken seriously. The Japanese Registry experience tells us that we are on the wrong path. We are not doing an adequate job of either self-appraisal or modification of practice. To do better, we must collect actionable metrics that affect clinically relevant results, self-assess our weaknesses without external agendas, and demonstrate better clinical outcomes than alternative treatment strategies.

## Funding Support and Author Disclosures

The author has reported that he has no relationships relevant to the contents of this paper to disclose.
